# Patient empowerment can lead to improvements in health-care quality

**DOI:** 10.2471/BLT.17.030717

**Published:** 2017-07-01

## Abstract

Frederico C Guanais argues that the expansion of primary care in the Americas is not enough to guarantee quality and effectiveness of health services. He talks to Andréia Azevedo Soares.

Q: How did you become interested in public health?

A: My father (Dr Oliveiros Guanais) was an anaesthesiologist and I heard a lot about medicine as I grew up. His favourite task at work was the interaction with patients at the pre-anaesthesia visit, when he put them at ease by listening to their stories and talking to them about the procedure to help improve the physiological and psychological results. But I studied civil engineering, and it was not until the end of the first year of my PhD in public policy that I became interested in public health, health policy and health services under the guidance of mentors such as James Macinko and Jan Blustein. At the time, there were so many exciting things going on in public health in Brazil: a community-oriented primary care programme (the Family Health Programme) was being scaled up nationally, a regulatory agency for health surveillance and a health economics unit were being set up, a generic drugs policy was being implemented, and Brazil’s – now highly acclaimed – HIV programme was just being rolled out. However, I was amazed by the lack of quantitative research on the extent to which the expansion of access to primary health care was improving health outcomes. I realized that I could contribute to such research and help to find better ways to deliver primary health programmes to scale.

Q: What drew you to the quality of health care as a research topic?

A: My initial health research was on the expansion of coverage of health services – a necessary first step in underserved areas. But I knew that unless services were delivered to a certain standard, increased coverage would not improve health outcomes. This is where the engineer in me got interested in solving the complex puzzles of health systems design and implementation, so that “health services for individuals and populations actually increase the likelihood of desired health outcomes” to quote the Institute of Medicine [in the USA] definition of quality of health care. I felt that the users’ perspective was missing and this led me to do research on health systems and health-care delivery from the patient’s perspective. In the last two years, a research group that includes colleagues from the IADB and other institutions published a series of articles based on the findings of surveys conducted in six countries in Latin America and the Caribbean, inspired by the international health surveys of the Commonwealth Fund, a New York-based think tank.

“I felt that the users’ perspective was missing.”

*Q: You published a study on this subject last year in the journal *Health affairs*. What did you find?*

A: This is one of the studies based on the IADB-financed surveys that I just mentioned. When the Commonwealth Fund looked at the health systems performance of 11 high-income countries, they found that 87% of patients in those countries rate the quality of care as “good”, “very good” or “excellent”. In our studies^,^ we replicated this exercise in middle-income countries across the Americas – Brazil, Colombia, El Salvador, Jamaica, Mexico and Panama – and we found that only about 40% of respondents rated the quality of care they received from the general practitioners as “good”, “very good” or “excellent”. Using the same data we have also found that, among the people who have a regular place of care, 40% of patients say doctors do not spend enough time with them, 26% say that doctors do not explain things in a way that they can understand, and about 36% say that doctors do not review their medications or discuss the potential side-effects. Moreover, our multivariate analysis shows that these variables, which are examples of quality from the patients’ perspective, are some of the best predictors of trust in the health system as a whole. This is a very important message to policy-makers: the experience patients have at primary care facilities is a strong predictor of how people perceive their national health system as a whole.

Q: Why did the expansion of health coverage in Brazil not go hand-in-hand with a high quality of care?

A: The Brazilian experience is important and representative for lower and middle-income countries that have rolled out universal health coverage (UHC). When the 1988 constitution introduced UHC, there was no implementation strategy and it was not until the expansion of the Family Health Programme (*Programa Saúde da Familia*) – now renamed the *Estratégia Saúde da Familia* (Family Health Strategy)^ – ^that many people in poorer areas had their first experience of Brazil's publicly funded health-care system (*Sistema Único de Saúde* or SUS). Thus the SUS’s main achievement was the piloting and national scale-up of the Family Health Programme during the 1990s and early 2000s. The Family Health Programme achieved spectacular results, especially in regions where coverage had been low, despite the poor quality of care.

Q: Why has Brazil failed to deliver a better quality of care overall since then?

A: When primary health care is extended to more people, they are grateful to have access to services but once they have access, they start thinking about quality. When services are provided on a large scale – in Brazil this primary care model serves 120 million people – the challenge is to create the managerial and organizational infrastructure capable of assuring a high quality of care. In 2011, Brazil launched a results-based financing scheme called the National Programme for Improvement of Access and Quality in Primary Care (PMAQ is the acronym in Portuguese) and its results may provide important insights into improving the quality of primary care at scale.

Q: What kind of reforms are there in Latin America aimed at improving the quality of care?

A: There is a strong consensus around the importance of improving the quality of primary care and most health ministries in the Americas are keen to pursue this goal through reforms. In the field, however, it’s been difficult to find the right strategies to implement such reform. Brazil, for example, is attempting to do so with the PMAQ, and I am hopeful that it will lead to positive results. But more innovation is needed in the primary care model to pursue such an agenda. For example, Colombia launched an important reform in the 1990s focused on coverage and financial protection rather than primary care. Now the private insurers that operate under the contributory insurance scheme have realized that the best way to address noncommunicable diseases (NCDs) is to invest in high-quality, patient-centered primary care as a way to improve health outcomes and control costs at the same time, which is a concept that applies to both public and private models. Chile has a strong primary care system that provides care of high quality, and recent reforms aim to improve access to after-hours care. Peru obtained excellent results with a primary care network focused on maternal and child health, but the country is seeking to improve its primary care services to address a broader range of conditions, especially noncommunicable diseases, aiming at both quality and efficiency – an ambitious but necessary approach. But again: it is one thing to have the political will to usher in reforms and another to know the best way to implement such reforms. This is the challenge. I think the best way is to take a bottom-up approach and to implement reform in consultation with patients.

Q: We saw a public uprising against poor public health services before the 2014 World Cup in Brazil. What can we learn from this episode?

A: It is rare to see dissatisfaction coupled with a grassroots movement demanding a better quality of health care. In Brazil's case, this demand was the seed of a popular movement that could have driven important changes in the way Brazil’s health systems are organized. But what we saw in Brazil was the emerging middle-class in the streets, fighting for better health services, rather than a broad mass movement demanding a better quality of services that would benefit all citizens. The SUS was built for everyone, but many affluent Brazilians buy private insurance and opt out, leaving the SUS for the poor. The 1988 constitution protects health as a universal right but in reality this right is not exercised equally.

Q: How important is quality of care to efforts to achieve the sustainable development goals (SDGs)?

A: It is always difficult to predict. The Millennium Development Goals and the SDGs are both focused on coverage not implementation, but coverage alone is not enough to guarantee quality. There is some discussion in some countries on how they might achieve universal coverage and, ultimately the SDGs, but very little discussion about the quality of care. We really need to focus on quality of care to achieve the SDGs. Quality of care is often a forgotten dimension. Coverage and quality of care must go hand in hand.

“We really need to focus on quality of care to achieve the SDGs. Quality of care is often a forgotten dimension.”

Q: Your research suggests that a more patient-centred approach to health-care delivery is the key to improving health-care quality. How willing are health professionals and managers to embrace this approach?

A: We are already moving away from the traditional doctor-knows-best approach. One of the drivers of this change is the rapid epidemiological transition from infectious to chronic diseases. If you get a flu shot, you are probably protected and the problem is solved. But noncommunicable diseases – such as diabetes and hypertension – are chronic problems and the patient needs to be involved in the health-care solution to achieve good results. Doctors also need to share their expertise and speak to patients in a way that they can understand. Latin America and the Caribbean are facing high levels of NCDs, particularly cardiovascular disease, diabetes, stroke, cancer and depression, and these will continue to be a major factor contributing to the need for patients' empowerment.

*Q: Are other factors driving *the need for patients' empowerment*?*

A: Yes, as discussed, public expectations are changing and this is clear when you compare the results of satisfaction surveys conducted at the entrance of clinics in countries in the Americas and in Africa. Patients in poorer, underserved parts of Africa are happy just to get an appointment, so a survey conducted at a clinic will overestimate satisfaction results because many people don’t even reach the facility. This used to be the case in the Americas, but now these patients expect more. This sense of growing public expectations about the quality of care is a positive thing and can lead to improvements.

